# The complete chloroplast genome of *Vandenboschia striata*, a common and widespread filmy fern (Hymenophyllaceae)

**DOI:** 10.1080/23802359.2021.2013744

**Published:** 2021-12-29

**Authors:** Ruonan Wang, Guohua Zhao, Zhen Wang, Zhongyao Lin, Xinyue Jiang, Shengde Lin, HokYan Chan, Yongfeng Hong, Yuli Zhang, Yingjuan Su, Ting Wang

**Affiliations:** a School of Life Sciences, Sun Yat-sen University, Guangzhou, China; b Fairy Lake Botanical Garden, Shenzhen & Chinese Academy of Sciences, Shenzhen, China; c Research Institute of Sun Yat-sen University in Shenzhen, Shenzhen, China; d College of Life Sciences, South China Agricultural University, Guangzhou, China

**Keywords:** *Vandenboschia striata*, chloroplast genome, phylogenetic analysis

## Abstract

*Vandenboschia striata* is a common and widespread filmy fern of Hymenophyllaceae. Its complete chloroplast genome is 147,014 bp in length, including a large single copy (LSC) region of 89,886 bp, a small single copy (SSC) region of 20,850 bp, and a pair of inverted repeats (IRs) of 18,139 bp. Totally, 132 genes are predicted in the cp genome embodies, including 88 protein coding genes, 36 tRNA genes, and eight rRNA genes. A maximum-likelihood tree was constructed to explore phylogenetic relationship. The result showed that *V. striata* was sister to *V.speciosa* with 100% bootstrap support. The complete chloroplast genome sequences of *V. striata* would be beneficial to further phylogenetic survey on classification of the related species or genera in Hymenophyllaceae.

*Vandenboschia striata* (D. Don) Ebihara, well-known as misapplied name *V. radicans* in China, is a filmy fern belonging to Hymenophyllaceae. The fern is only 15–40 cm tall with a deep smoke-colored rhizome. Stipes are light brown and dark green-brown veins with dichotomous raised on each surface (Liu et al. 2013). As a common and widespread filmy fern, *V. striata* distributes in China (Guangdong, Guangxi, Guizhou, Hainan, Sichuan, Taiwan, and Yunnan), Bjutan, India, Japan, Laos, Myanmar, Nepal, and Vietnam (Liu et al. 2013). The plant prefers to grow on wet rocks near streams and slopes with altitudes 400–2700 m. Currently, the genera classification of Hymenophyllaceae, especially *Vandenboschia*, is greatly controversial (PPG I 2016). In addition, there are also problems involving in fern complex and hybridization such as *V. radicans* complex (Ebihara et al. 2005; Ebihara et al. 2009). Hence, sequencing of the complete chloroplast genome of *V. striata* will greatly contribute to solving these issues.

*Vandenboschia striata* was collected from Fairy Lake Botanical Garden, Shenzhen (22°35′8.39″N, 114°11′6.68″E), which was immediately treated with liquid nitrogen, and stored in the −80 °C. The specimen is deposited at the Herbarium of Sun Yat-sen University (Wang Ruonan: wangruonan0630@163.com and voucher number: Wang202101). We extracted the genomic DNA from approximate 100 mg fresh and young leaves using Tiangen Plant Genomic DNA Kit (Tiangen Biotech Co., Beijing, China), which was further fragmented into 300 bp. After the Illumina library was constructed, sequencing was performed on a Hiseq 2500 platform (Illumina Inc., San Diego, CA). About 2.93 Gb paired-end data were generated and 2.67 Gb high-quality clean data were further assembled using GetOrganelle (Jin et al. 2020). Protein coding genes, rRNA genes, and tRNA genes were separately annotated through PGA (Qu et al. 2019) and tRNAscan-SE programs (Lowe and Eddy 1997), followed by adjustment and confirmation of codon and intron/exon boundaries using Geneious v8.1 (Kearse et al. 2012). Upload GenBank number was MZ911852. Raw reads were deposited in the GenBank Sequence Read Archive (SRA: SRR15564830).

The complete chloroplast genome of *V. striata* is 147,014 bp in length, with an overall GC content of 37.5%. It demonstrates a typical quadripartite circular structure, involving in a large single copy (LSC) region of 89,886 bp, a small single copy (SSC) region of 20,850 bp, and a pair of inverted repeats (IRs) of 18,139 bp. Totally, 132 genes are predicted in the cp genome embodies, including 88 protein-coding genes, 36 tRNA genes, and eight rRNA genes. Three genes (*clpP*, *rps12*, and *ycf3*) contain two introns, and 11 genes (*atpF*, *ndhA*, *ndhB*, *rpl2*, *rpoC1*, *rps16*, *trnA*-UGC, *trnI*-GAU, *trnG*-UCC, *trnL*-CAA, and *trnV*-UAC) have one intron, whereas 13 genes (*psbA*, *rps7*, *rps12*, *trnA*-GUU, *trnI*-GAU, *trnL*-GAU, *trnN*-GUU, *trnR*-ACG, *trnV*-GAC, *4.5S rRNA*, *5S rRNA*, *16S rRNA*, and *23S rRNA*) have two copies.

To investigate the phylogenetic relationship of *V. striata*, we downloaded 11 fern cp genomes from the NCBI database. These complete chloroplast genome sequences were aligned using MAFFT with default parameter (Katoh and Standley 2013), then a maximum-likelihood tree was constructed using RAxML v.8.2.12 with *Osmundastrum cinnamomeum* (KF225592) as outgroup and 1000 replicates (Stamatakis 2014). As a result, the phylogenetic tree showed that *V. striata* was sister to *V. speciosa* (Figure 1). The complete chloroplast genome sequences of *V. striata* would be beneficial to the further phylogenetic survey on the classification of the related species or genera in Hymenophyllaceae.

**Figure 1.**
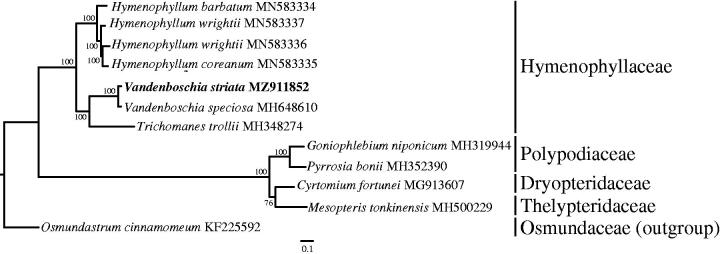
Maximum-likelihood phylogenetic relationship of *Vandenboschia striata* and other 11 ferns including *Osmundastrum cinnamomeum* as outgroup based on whole chloroplast genome sequences. The bootstrap values are shown on each node.

## Data Availability

The data that support the findings of this study are openly available in GenBank of NCBI at https://www.ncbi.nlm.nih.gov/nuccore/MZ911852, GenBank accession number MZ911852. Raw sequencing reads in this study are deposited in https://www.ncbi.nlm.nih.gov/sra/PRJNA756937, with SRA number SRR15564830.
